# A physical model inspired density peak clustering

**DOI:** 10.1371/journal.pone.0239406

**Published:** 2020-09-24

**Authors:** Hui Zhuang, Jiancong Cui, Taoran Liu, Hong Wang

**Affiliations:** 1 School of Information Science and Engineering, Shandong Normal University, Jinan, China; 2 Shandong Provincial Key Laboratory for Distributed Computer Software Novel Technology, Shandong Normal University, Jinan, China; Torrens University Australia, AUSTRALIA

## Abstract

Clustering is an important technology of data mining, which plays a vital role in bioscience, social network and network analysis. As a clustering algorithm based on density and distance, density peak clustering is extensively used to solve practical problems. The algorithm assumes that the clustering center has a larger local density and is farther away from the higher density points. However, the density peak clustering algorithm is highly sensitive to density and distance and cannot accurately identify clusters in a dataset having significant differences in cluster structure. In addition, the density peak clustering algorithm’s allocation strategy can easily cause attached allocation errors in data point allocation. To solve these problems, this study proposes a potential-field-diffusion-based density peak clustering. As compared to existing clustering algorithms, the advantages of the potential-field-diffusion-based density peak clustering algorithm is three-fold: 1) The potential field concept is introduced in the proposed algorithm, and a density measure based on the potential field’s diffusion is proposed. The cluster center can be accurately selected using this measure. 2) The potential-field-diffusion-based density peak clustering algorithm defines the judgment conditions of similar points and adopts different allocation strategies for dissimilar points to avoid attached errors in data point allocation. 3) This study conducted many experiments on synthetic and real-world datasets. Results demonstrate that the proposed potential-field-diffusion-based density peak clustering algorithm achieves excellent clustering effect and is suitable for complex datasets of different sizes, dimensions, and shapes. Besides, the proposed potential-field-diffusion-based density peak clustering algorithm shows particularly excellent performance on variable density and nonconvex datasets.

## 1. Introduction

Clustering is an important task in data mining. Exploring data clustering is important to understand the features of any given data, the relationship between these data, and the overall data structure [1]. Cluster analysis has played important roles in bioscience, social networks, and web analysis. For example, in protein interaction data, important protein cluster structures can be detected using clustering methods; this aids medical professionals in finding comorbid or new disease subtypes [2]. In social networks, clusters can be used to determine groups that frequently communicate and understand communication within and between communities to reduce network overload [3]. In sensor networks, closely related nodes can be identified via clustering, and accordingly the applied information collection algorithm can be optimized [4]. Clustering analysis is a critical process that has attracted significant research attention and rapidly progressed; some examples of such analysis are k-means [5], k-medoids [6], CURE [7] and BIRCH [8], DBSCAN [9], OPTICS [10], WaveCluster [11], STING [12], statistical clustering [13], spectral clustering [14], subspace clustering [15], integrated clustering [16], and graph-based clustering [17]. Besides, there is automatic clustering that introduces a metaheuristic algorithm, which can automatically determine the optimal number of clusters. However, the problem of finding the optimal number of clusters is NP-Hard [18]. Real data have different structures; thus, research on clustering is extremely challenging.

In 2014, Rodriguez proposed the DPC (clustering via fast search and finding density peaks) algorithm [19] that exploits the advantages of both density and distance based clustering methods. On the one hand, similar to the k-medoids algorithm, DPC clustering depends on only the distance between samples and requires fewer parameters to be adjusted. On the other hand, identical to the DBSCAN algorithm, the DPC algorithm can find nonspherical clusters. In addition, the DPC algorithm is similar to the mean-shift algorithm [20] in selecting cluster centers, which can determine the number of clusters automatically. Compared with the mean-shift algorithm, DPC does not need to maximize the density of each sample; thus, the algorithm is simple and effective.

Although the DPC algorithm [19] has significant advantages over other clustering algorithms, many areas require improvement. For example, the cluster center selection method is too sensitive to distance and local density, incorrect cluster center points can easily be selected, and it cannot adapt to differences in the data structure. Specifically, for datasets with significant differences in cluster density, correct cluster centers cannot be effectively identified. In addition, the allocation strategy of noncentral points excessively relies on the nearest higher density points, thus being prone to attached allocation errors. In particular, each data point is classified into a cluster from among clusters that are closest to it and have high local density. If the allocation of this data point is incorrect, the allocation of subsequent data points will also be incorrect; thus, the correct cluster will not be obtained.

To solve the above problems, this study proposes a PFD-DPC (potential-field-diffusion-based density peak clustering) algorithm. Based on the potential field concept, the density measure is redefined, and reasonable cluster centers are selected. Accordingly, the distribution method of noncentral points is optimized, improving the clustering accuracy and efficiency. The primary contributions of this study are summarized as follows.

1. A new density measure based on the diffusion of the potential field is proposed to make the selection of cluster center points more reasonable, thus solving the problem of easily selecting incorrect cluster centers.
2. An improved noncentral point allocation strategy is proposed to avoid the allocation strategy from relying excessively on the nearest higher density points, which solves the problem that the traditional DPC algorithm is prone to attached allocation errors.
3. This study conducted many experiments on synthetic and real-world datasets to verify the performance of the proposed PFD-DPC algorithm, and the experimental results demonstrate that the obtained clustering effects are significantly better than those of the traditional clustering algorithm and other DPC-improved algorithms.

The structure of this paper is as follows. Section 2 summarizes the related work of DPC algorithm; Section 3 outlines the idea and process of DPC algorithm; Section 4 proposes PFD-DPC algorithm, including the proposal of potential, relevant definitions, cluster center selection method and non-central point allocation algorithm; Section 5 verifies the performance of the PFD-DPC algorithm through a large number of experiments and discusses it; Section 6 discusses the sensitivity and running time of the algorithm; Section 7 summarizes PFD-DPC algorithm and point out the improvement of the algorithm and the future research direction.

## 2. Related work

Since the DPC algorithm was first proposed, studies have attempted to continuously improve its performance. Improvements to the DPC algorithm are primarily reflected relative to the following four aspects.

The first aspect of these is the improvement in local density and relative distance. The method to improve local density is to find an effective kernel method to calculate local density. These kernels help selecting cluster centers and reducing the dependence of the DPC algorithm on cutoff distance. Previously, Mehmood [21] proposed a nonparametric DPC algorithm based on thermal diffusion (CFSFDP-HD). This algorithm is based on the kernel density estimation technology of statistics, i.e., the contribution of each point to the total density function is expressed by the kernel function, and the total density function is the sum of the influence functions of each point. Here, the optimal bandwidth of the kernel function can be obtained; thus, the sensitivity of the algorithm to the *dc* parameter value is reduced. The most significant problem with such methods is the high cost of calculation because the density of each point should be explicitly calculated, and this density is the sum of the contribution of the density function of all points, which limits the applicability of the algorithm to large-scale applications.

To deal with uneven density classes and reduce the impact of the *dc* parameter, a DPC clustering method based on the neighbor concept has been previously proposed. This kind of method assumes that considering the contribution of all points is not necessary for calculating the local density of any point, i.e., only the contribution of points around the data point must be considered. They use local information to calculate the local density of data points. Consequently, this algorithm reduces the calculation of the similarity matrix and enhances the local density’s perception of the context of the data points. However, when the density difference between clusters is significant, local density substantially impacts the cluster centers. Du [22] proposed DPC based on k-nearest neighbors (DPC-KNN) and introduced the k-nearest neighbors concept into the DPC algorithm. This algorithm also uses PCA dimensionality reduction to solve the problem of clustering high-dimensional data. Juanying [23] stated that using an exponential kernel to measure the local density of samples in the traditional DPC algorithm is better than directly estimating the local density of samples. However, the exponential kernel measurement method is too sensitive to cutoff distance; thus, she proposed a DPC algorithm based on optimizing the local density of k-nearest neighbors (KNN-DPC). In addition, Yaohui [24] proposed the adaptive DPC algorithm based on the k-nearest neighbor and aggregation strategy. However, using this method, the manner in which an appropriate K value is selected is very important. If K is too small, the local density is susceptible to noise interference. In contrast, if K is too large, the local density may be inaccurate because the k-nearest neighbors may contain non-nearest neighbors.

To solve the problem of over-dependence on K, Rui [25] proposed a shared-nearest-neighbor-based clustering algorithm via fast search and finding density peaks, i.e., (shared-nearest-neighbor density peak clustering; SNN-DPC). This algorithm considers the first-order and shared neighbors of data points, redefines the local density and the distance between the nearest higher density points, and proposes a two-step allocation strategy. In addition, Li [26] used the fuzzy neighborhood relationship to calculate the local density of data points and used the comparison distance to replace the distance of the nearest higher density points in the DPC algorithm.

The second aspect is the automatic determination of the number of clusters and cluster centers.

Most methods used to automatically determine cluster centers are based on the overall structure. Wu [27] proposed the ADPC algorithm, redefined the decision value equation, proposed an adaptive cutoff distance adjustment method based on the Gini coefficient, and established a mechanism to automatically obtain cluster centers without manually specifying the cluster centers and cutoff distance. However, the occurrence of attached allocation errors and ignoring low-density clusters on datasets having significant density differences is easy when using this algorithm.

Tao [28] proposed a density peak clustering algorithm to automatically determine clustering centers. First, that algorithm constructs a *γ* sorting graph according to the DPC method, and then potential cluster centers are determined based on the inflection points in the sorting graph. Finally, the actual cluster centers are screened from the potential cluster centers. According to the literature [29], the normalized local density and distance to the point with high density can improve clustering results. He normalized the obtained local density and distance to the point with high density, and then identified clustering centers. In addition, Liang [30] applied the divide and rule strategy to automatically identify cluster centers. This method belongs to the global calculation method and does not require prior knowledge to adjust parameters.

The third aspect is the allocation strategy of noncentral points. Improvements in the noncentral point allocation method attempts to develop a new method to assign noncentral points to clusters more accurately.

Bie [31] proposed the Fuzzy-CFSFDP algorithm, which considers points higher than the average value of the local density estimation as the local clustering centers. Therein, data points are assigned to clusters to which the nearest local cluster center belongs, and then clusters with close peak density and average density at the edge of the cluster are merged.

Qiu [32] proposed a clustering algorithm based on nearest neighbor descent, which organizes data points into fully connected graphs, and then uses the nearest neighbor descent algorithm to map the graph to a tree. According to this tree, each data point is connected to its nearest neighbor only in the direction of density decline. In a previous study [23], a two-step allocation strategy was proposed. In that strategy, a connected graph is constructed based on the KNN, beginning with all types of cluster centers using breadth-first search to allocate noncentral points. Then, the KNN majority voting strategy is used to allocate noncentral points. Accordingly, the fuzzy weighted KNN algorithm based on the density DPC points (FKNN-DPC) [33] was proposed, wherein noncentral points are allocated using the fuzzy weighted KNN method. Lotfi [34] proposed IDPC, which uses the label propagation method to distribute the remaining points according to Euclidean distance. Similarly, the DPC-DLP clustering algorithm [35] first constructs weighted all connected graph, and the weight on an edge is the KNN kernel distance. Thus, tag propagation based on random walk is realized.

The fourth aspect involves new application scenarios of the DPC algorithm. Research on DPC algorithm applications attempts to apply the DPC algorithm and its improved versions to various real-world tasks to solve practical problems.

For example, Mehmood [36] applied the DPC algorithm in the biomedical field. The DPC algorithm was run on a leukemia dataset to identify B-lineage acute lymphoblastic leukemia (ALL), T-lineage ALL, and acute myeloid leukemia with an accuracy of 97.3684%. The DPC algorithm was also run on a breast cancer dataset to distinguish drug-resistant and sensitive subclasses, showing an accuracy of 70.8333%.

In addition, Shi [37] applied the DPC algorithm to scene image clustering, and Chen [38] used the DPC algorithm to estimate the age range of a given facial image. In a previous study [39], Zhang used a density peak clustering algorithm to extract the abstracts of multiple documents. Wang applied the DPC algorithm and information entropy to detect and eliminate noise features in datasets [40].

Shi [37] and Bai [41] implemented the DPC algorithm on an overlapping community partition problem. Herein, a new distance matrix is defined to overcome the defect of the integer adjacency matrix and the probability that each point belongs to a different cluster is given, so as to achieve the goal of dividing overlapping communities.

The above are the improved DPC algorithms that the authors know. There is no idea of introducing the concept of potential field and potential field diffusion into density peak clustering. At the same time, for the definition of similar points, the proposed PFD-DPC algorithm is also different from the above algorithms.

## 3. Density peak clustering algorithm

Currently, the DPC algorithm is widely used. Rodriguez proposed the DPC algorithm in *Science* in 2014. The DPC algorithm is based on two assumptions: the local density of points around cluster centers is relatively low, and the distance between cluster centers is relatively large. Accordingly, the DPC algorithm proposes two measures to describe the density and distance of data points *i*, i.e., local density *ρ*_*i*_ and distance to the nearest high density point *δ*_*i*_.

In terms of *ρ*_*i*_, local density is measured in terms of two parameters: cutoff distance and Gaussian cutoff distance is given in Eq (1).

$$\rho_{i}=\sum_{i\neq j}\chi (d_{ij}-d_{c})$$ (1)

Here, *d*_*ij*_ is the Euclidean distance between data points *i* and *j*, and *dc* is a custom cutoff distance.

Function *χ*(*x*) is given in Eq (2).

$$\chi (x)=\left\{ \begin{array}{c} 1\;x<0\\ 0\;x\geq 0 \end{array}\right.$$ (2)

When the Euclidean distance between data points *i* and *j* is less than the cutoff distance, the function value is 1, which increases the value of local density by 1, and when the Euclidean distance between data points *i* and *j* is greater than or equal to the cutoff distance, the function value is 0. Therefore, the local density calculated by cutoff distance is the number of data points within the cutoff distance of a given data point.

The definition of Gaussian kernel distance is given in Eq (3).

$$\rho_{i}=\sum_{i\neq j}e^{\left[-{\left(\frac{d_{ij}}{d_{c}}\right)}^{2}\right]}$$ (3)

Here, the terms have the same definitions as those in Eq 1.

Eqs (1) and (2) show that local density *ρ* is positively correlated with the number of points in the *dc* neighborhood to *i*. The difference between cutoff distance and Gaussian kernel distance is that local density *ρ* represented by the former is discrete, and local density *ρ* represented by the latter is continuous.

Relative to *δ*_*i*_, the distance to the nearest high density point is calculated as shown in Eq (4).

$$\delta_{i}=\underset{j:\rho_{j}>\rho_{i}}{\min}\left(d_{ij}\right)$$ (4)

Here, *ρ*_*i*_ and *ρ*_*j*_ are the local density of data points *i* and *j*, and *d*_*ij*_ is the Euclidean distance between data points *i* and *j*.

For the point with the highest local density, the distance of the nearest higher density point *δ*_*i*_ is defined in Eq (5).

$$\delta_{i}=\underset{i\neq j}{\max}\left(\delta_{j}\right)$$ (5)

Here, the point with the highest local density also has the largest *δ* value.

To better represent cluster centers, the DPC algorithm calculates the decision value *γ*_*i*_ of each data point *i* as follows.

$$\gamma_{i}=\rho_{i}\times \delta_{i}$$ (6)

This indicates that the decision value of a data point is the product of its local density and the distance to the nearest higher density point.

The definition of a boundary point by the DPC algorithm is described as follows. If the distance between an allocated data point and a point in another cluster is less than cutoff distance *dc*, the allocated data point is considered a boundary point.

Based on the above definitions, the DPC algorithm proposes a clustering process involving three steps: finding clustering centers, assigning noncentral points to corresponding clusters, and processing boundary points.

In the first step, the DPC algorithm calculates the local density and the distance to the nearest higher density point *δ* for each data point, and then calculates decision value *γ* based on the calculated *ρ* and *δ*. Then, the DPC algorithm plots a decision graph. In this graph, the abscissa is *ρ*, the ordinate is *δ*, and data points with high values of *ρ* and *δ* are selected as cluster centers.

In the second step, after selecting the cluster centers of the sample, the data points are arranged in descending order of local density, and the remaining data points are attributed to the cluster of the nearest high local density data points.

In the third step, the highest local density of the boundary points is deemed the threshold. If the local density of the data point is greater than or equal to the threshold, the data point is considered the core point of the cluster. Otherwise, the data point is considered a noise point.

**Fig 1:**
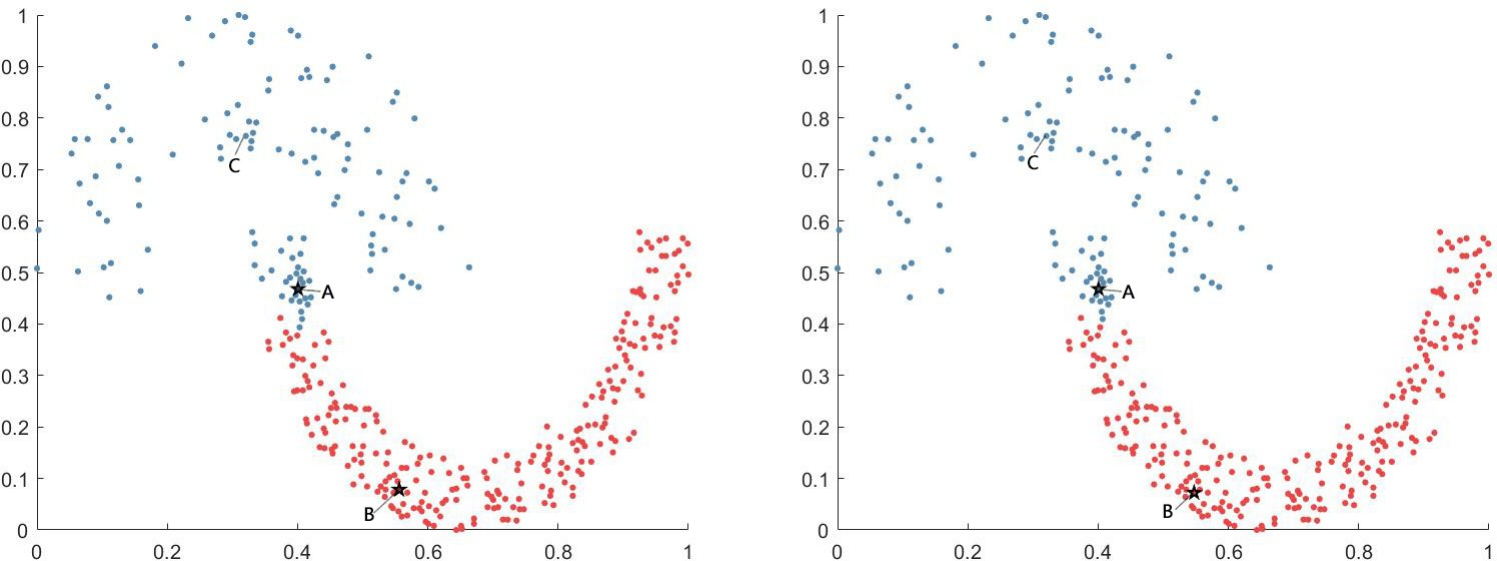
The DPC algorithm on Jain dataset. (A) use cutoff distance and (B) use kernel distance.

Although the experimental results demonstrate that the DPC algorithm can obtain better clustering results in many cases, its shortcomings are significant. For some datasets, the DPC algorithm cannot find the correct clustering center, and even in the case of datasets for which it obtains the clustering center, the result may be incorrect. Consider the Jain dataset as an example. The DPC algorithm exhibits poor clustering effect on this dataset. Fig 1 shows the clustering results of the DPC algorithm on the Jain dataset.

Here, points A and B are the cluster centers obtained by the DPC algorithm, and point C is the real cluster center of the low-density cluster. As can be observed in Fig 1, regardless of the cutoff or kernel distances, the DPC algorithm does not select the correct cluster center, resulting in incorrect data point allocation because the Jain dataset is a variable density dataset. On one hand, points in low-density clusters typically have small *ρ* values, and even large *δ* values cannot effectively improve their inferior position in decision graphs. On the other hand, points in high-density clusters tend to have high *ρ* values. Compared to the points in low-density clustering, selecting these points as clustering centers is easy.

**Fig 2:**
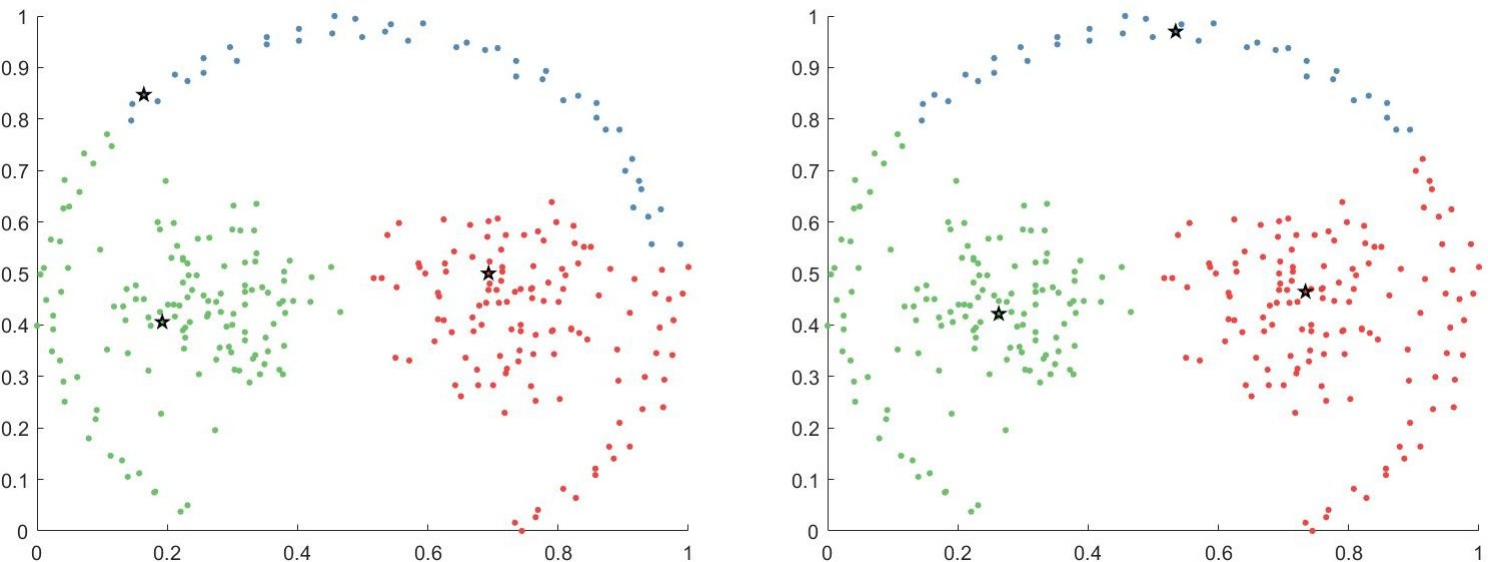
The DPC algorithm on Pathbased dataset. (A) use cutoff distance and (B) use kernel distance.

In the following sections, this paper considers the Pathbased dataset as another example. Fig 2 shows the clustering results of the DPC algorithm when implemented on the Pathbased dataset.

Here, two points can be observed. First, the DPC algorithm can correctly identify the cluster centers. Second, in the initial allocation process, noncentral points are allocated to the correct clusters. However, the points on both sides of the ring are assigned to the incorrect cluster owing to the DPC algorithm’s three-step allocation strategy. When a noncentral point is assigned to an incorrect cluster, subsequent points will also be assigned to the incorrect cluster, resulting in inaccurate results.

In summary, the DPC algorithm must be improved relative to cluster center selection and noncentral point allocation.

## 4. Potential-field-diffusion-based density peak clustering algorithm

In consideration of the above problems, this paper proposes the PFD-DPC algorithm. First, the PFD concept is proposed, following which a new measurement of *ρ* is presented. Then, the PFD-DPC algorithm is described, including the clustering center selection and noncentral point allocation algorithms.

### 4.1 Potential field diffusion principle

This part will introduce the concept of potential field and influence propagation.

#### 4.1.1 Potential field

According to Newton’s law of universal gravitation, each object has gravitation: a large mass results in strong gravitation, and the longer the distance, the smaller is the gravitation.

Assume *m*_*i*_ and *m*_*j*_ are two particles in space (particles are points where volume does not exist, but mass does exist). According to the law of universal gravitation, gravitation between particles *m*_*i*_ and *m*_*j*_ can be expressed as follows.

$$F=G\times \frac{m_{i}\times m_{j}}{r_{ij}\times r_{ij}}$$ (7)

Here, *G* is the constant of gravity, and *r*_*ij*_ is the Euclidean distance between particles *m*_*i*_ and *m*_*j*_.

For clustering purposes, authors simplify Eq (7) [42]. First, this paper considers that all points in the data space follow Newton’s law of universal gravitation and that the mass of all data objects is unit mass 1. Second, this paper considers that object nodes *i* and *j* are high-dimensional data points; thus, they should be expressed in vector form, i.e., $\vec{r_{i}}$ and $\vec{r_{j}}$. Therefore, $\vec{r_{ij}}=\vec{r_{i}}-\vec{r_{j}},\hat{r_{ij}}=\frac{\vec{r_{ij}}}{r_{ij}}$ can be obtained, where *r*_*ij*_ is the Euclidean distance between two objects. The gravity of nodes *i* and *j* is expressed as follows.

Fij→(rij→)=Grij^rij2 (8)

Third, a threshold value *ε* is set to modify Eq (8) to avoid the singular value of the equation when the *r*_*ij*_ infinity is close to zero. The gravity of modified nodes *i* and *j* is expressed as follows.

Fij→(rij→)={Grij^rij2rij≥ε0rij<ε (9)

Finally, this paper sets the value of *G* to 1 because *G* is the gravity constant, which is barely relevant clustering data objects. Therefore, gravity is expressed as follows.

Fij→(rij→)={rij^rij20rij≥σrij<σ (10)

**Definition 1.** Cumulative potential

The cumulative potential of data object *i* is the sum of the attractiveness of all data objects in the data space to *i*, as shown in Eq 11.

$$\Phi_{i}=\sum_{j\neq i}\overset{\to }{F_{ij}}(\overset{\to }{r_{ij}})$$ (11)

Note that *ε*, which is introduced to avoid the singular value problem, is a hyper parameter, and its optimal value is experimentally obtained.

#### 4.1.2 Influence propagation

According to the influence propagation principle of complex networks, the influence probability of nodes on other nodes is related to the degree of nodes [43]. Herein, this paper expresses a network as *G* = (*V*,*E*,*W*), where *V* is the set of nodes, *E* is the set of relationships among nodes, and *W* is the relationship matrix of network *G*. The influence probability of nodes in *G* can be used to measure the influence propagation of nodes. The element *p*_*ij*_ of *G*′*s* influence probability matrix *P* represents the one-step influence probability of node *i*∈*V* on node *j*∈*V*. *p*_*ij*_ is calculated as follows.

$$p_{ij}=\frac{w_{ij}}{\sum_{k\in nbs(i)}w_{ik}}$$ (12)

Here, *w*_*ij*_ is an element in row *i* and column *j* of the matrix *W*, and *nbs*(*i*) is the neighbor set of the node *i*.

In network *G*, the two-step influence probability matrix between nodes can be expressed as the product of two one-step influence probability matrices. When further considering attenuation factors in the process of information dissemination, the influence propagation process can be formalized as follows.

Following one influence propagation step, the node relationship matrix is *WβP*.

After two influence propagation steps, the node relationship matrix is *WβP*^2^.

…

After *k*−1 influence propagation steps, the node relationship matrix is *WβP*^*k*−1^.

Here, *β* is the attenuation coefficient of influence propagation.

Each element of the above relationship matrix represents a proximity between nodes in the network, which is actually the embodiment of the number of nodes in the influence propagation process. Therefore, the sum of the above relation matrix represents node proximity after *k*−1 influence propagation fusion steps. The fused relation matrix is presented in Eq (13).

$$W_{\Sigma }=W(\beta P+\beta^{2}P^{2}+\cdots +\beta^{k-1}P^{k-1})$$ (13)

Here, the parenthesized expression in Eq (13) is the Katz similarity index [44]. Because *k* can tend to infinity, the Katz similarity index belongs to the global similarity in a complex network. Therefore, the influence propagation calculated based on Eq (13) is the global influence of the node on network *G*.

The above potential field and diffusion concepts produce the following inferences. As the potential field of a data point increases, the core of the node grows stronger, and the potential spread can be based on both local and global data distribution information. Selecting a clustering center in this manner can achieve better clustering results; therefore, this study proposes a clustering algorithm based on PFD.

### 4.2 Definitions

Here, this paper presents several definitions.

**Definition 1:** k-nearest neighbors. For any point *i* in dataset *S*, its k-nearest neighbors are expressed as *σ*(*i*).

**Definition 2:** Common neighbor. For any points *i* and *j* in dataset *S*, their common neighbors are the intersection of their k-nearest neighbor sets, which is expressed as follows.

ω(i,j)=σ(i)∩σ(j) (14)

**Definition 3:** Potential field *θ*(*i*,*j*).

The potential field of nodes *i* and *j* is presented in Eq (15), which is the cumulative potential of the common neighbors of nodes *i* and *j*.

$$\theta (i,j)=\left\{ \begin{array}{cc} \frac{{\left|\omega (i,j)\right|}^{2}}{\sum_{p\in \omega (i,j)}\left({d_{ip}}^{2}+{d_{jp}}^{2}\right)}, & i,j\in \omega (i,j)\\ 0, & else \end{array}\right.$$ (15)

**Definition 4:** PFD similarity *Θ*(*i*,*j*).

The PFD of nodes *i* and *j* is expressed in Eq (16), which represents k-step diffusion of the potential field between nodes *i* and *j*.

$$\Theta (i,j)=\left\{ \begin{array}{cc} \frac{{\left|\omega (i,j)\right|}^{2}\times \sum_{m\in \sigma (i)}\sum_{n\in \sigma (m)}\ldots \sum_{q\in \sigma (o)}\left({d_{im}}^{2}+{d_{mn}}^{2}+\ldots +{d_{oq}}^{2}\right)}{\sum_{p\in \omega (i,j)}\left({d_{ip}}^{2}+{d_{jp}}^{2}\right)}, & i,j\in \omega (i,j)\\ 0, & else \end{array}\right.$$ (16)

Here, *d*_*im*_, *d*_*mn*_, *d*_*oq*_, *d*_*ip*_, and *d*_*jp*_ are the Euclidean distances between points *i* and *m*, *m* and *n*, *o* and *q*, *i* and *p*, and *j* and *p*, respectively. The PFD similarity is calculated when points *i* and *j* are k-nearest neighbors to each other; otherwise, the PFD similarity between the two is zero.

One term in Eq (16) is as follows.

$$\sum_{m\in \sigma (i)}\sum_{n\in \sigma (m)}\ldots \sum_{q\in \sigma (o)}\left({d_{im}}^{2}+{d_{mn}}^{2}+\cdots +{d_{oq}}^{2}\right)$$

**Fig 3:**
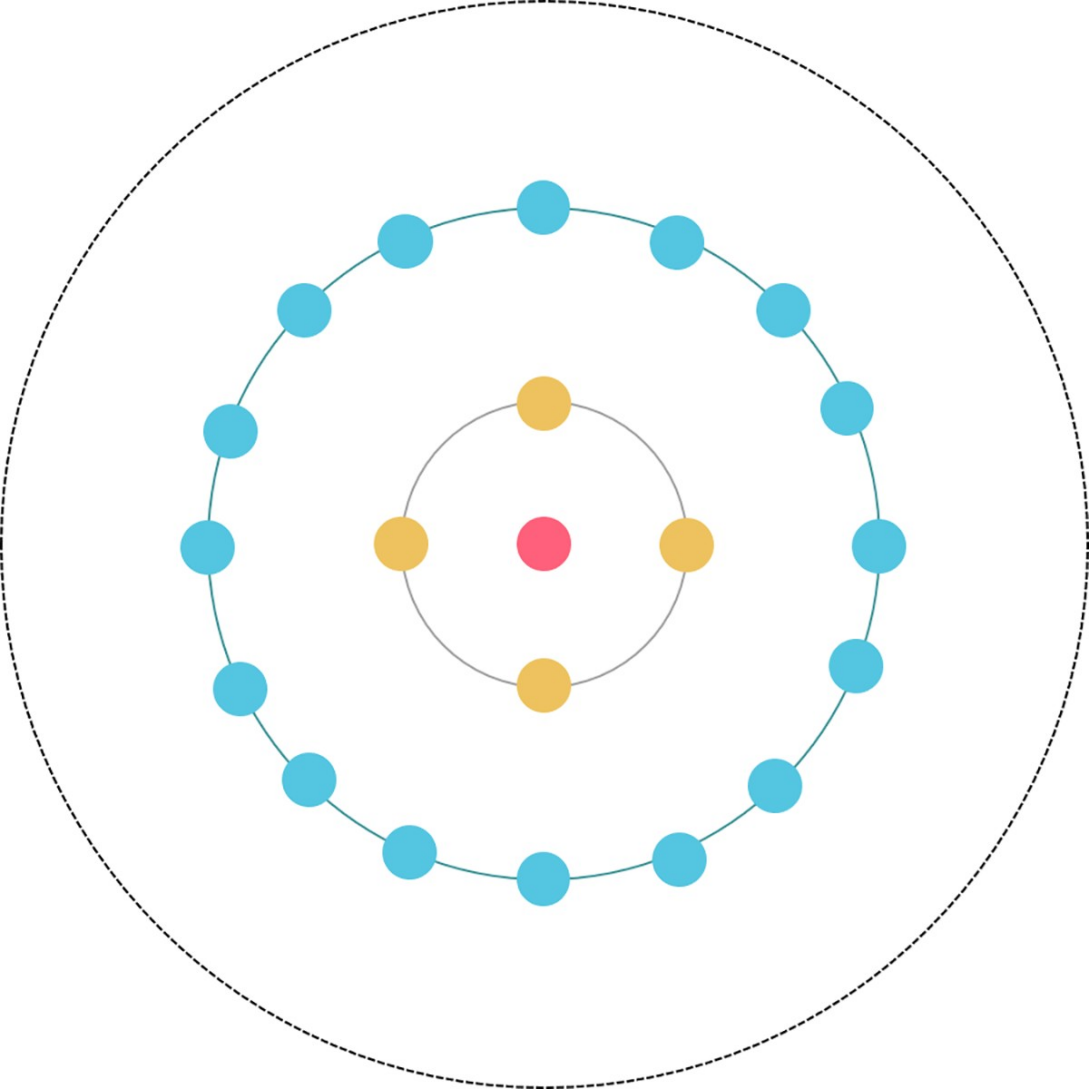
k-layer diffusion of potential field.

This expression can be more intuitively represented as shown in Fig 3 (assuming the number of neighbors of each data point is four), i.e., the potential field of the red layer diffuses to the yellow layer, and then to the blue layer via the yellow layer and so on and so forth, finally reaching the black layer (layer *k*).

**Definition 5:** Local density. For any data point *i* in dataset *S*,*T*(*i*) = {*t*_1_,*t*_2_,…,*t*_*k*_} denotes the set of the first *K* points with the highest PFD similarity to data point *i*. Here, the local density of point *i* is defined as the sum of the PFD similarity of each element in *T*(*i*), which can be expressed as follows.

$$\rho_{i}=\sum_{j\in T(i)}\Theta (i,j)$$ (17)

**Definition 6:** Distance to nearest higher density point. For any data point *i* in dataset *S*, if a point *i* that satisfies *ρ*_*j*_>*ρ*_*i*_ is found, find the closest point to data point *i* in the set of all *j* points that satisfy this condition, and use the minimum value of this distance as *δ* value of point *i*. It is expressed as follows.

$$\delta_{i}=\underset{j:\rho_{j}>\rho_{i}}{\min}d_{ij}$$ (18)

For data points with the highest local density, the *δ* value is defined as the largest *δ* value in the sample, which is expressed as follows.

$$\delta_{i}=\underset{j\in (T-i)}{\max}\left(\delta_{j}\right)$$ (19)

**Definition 7:** For any data point *i* in dataset *S*, its decision value *γ* is the product of the local density *ρ* and its nearest higher density point distance *δ*.

$$\gamma_{i}=\rho_{i}\times \delta_{i}$$ (20)

The decision value can be used to quickly select cluster centers.

**Definition 8:** Similar point. For any data points *i* and *j* in dataset *S*, if the relationship between points *i* and *j* satisfies Eq (21), then point *j* is referred to as a similar point of point *i*.

$$\{y|y\in \sigma (i)\cap p\in \sigma (j)\}>\left\lceil \frac{K+1}{2}\right\rceil$$ (21)

If two points have more common nearest neighbors, the more similar the two data points are. Therefore, this paper stipulates that if the number of common nearest neighbors of two data points is greater than $\left\lceil \frac{K+1}{2}\right\rceil$, the two points are called similar points. That is to say, no matter whether the value of K is odd or even, the number of common neighbors of two similar points is always more than half of the number of K nearest neighbors. If this paper lowers the standard of the number of common nearest neighbors of similar points, the accuracy of allocation will be reduced in the subsequent allocation of data points, and if this paper raises the standard of the number of common nearest neighbors of similar points, the operation amount of the algorithm will be increased.

Eq (21) can also be expressed as follows.

$$|\omega (i,j)|>\left\lceil \frac{K+1}{2}\right\rceil$$ (22)

### 4.3 Determining the number of clusters

**Fig 4:**
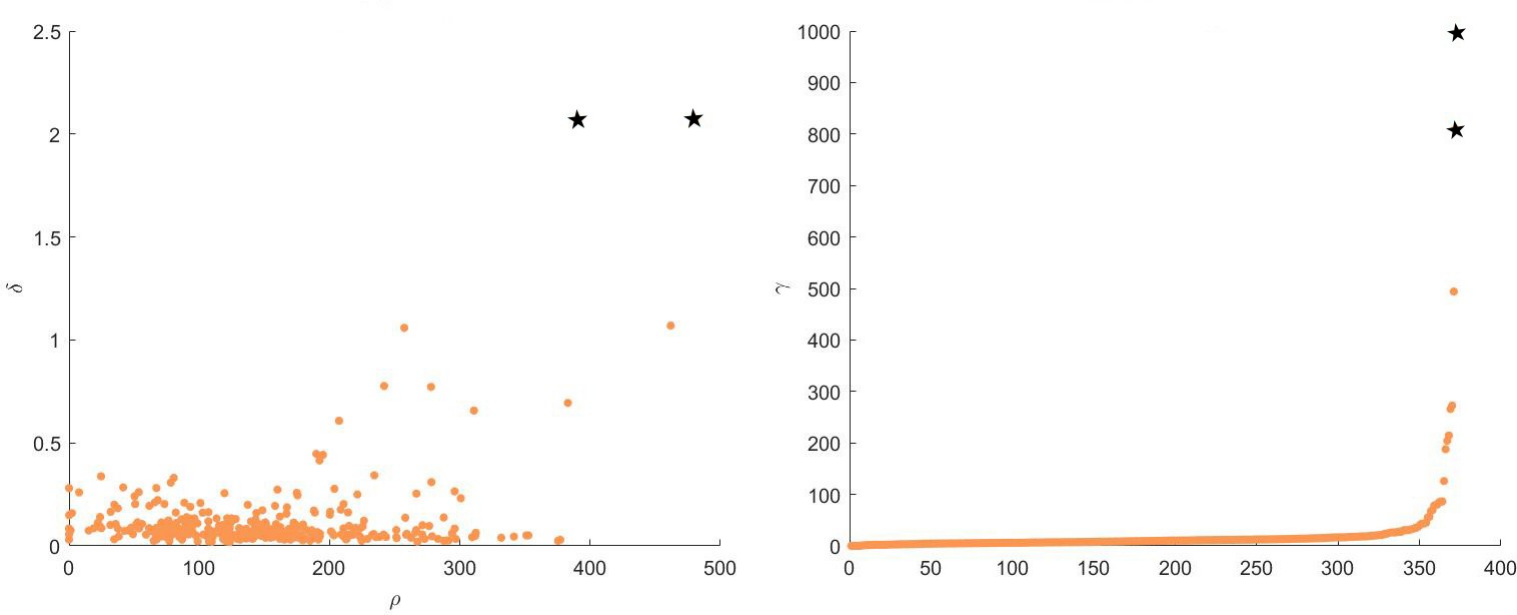
Decision graph. (A) ρ−*δ* graph and (B) *γ* graph.

The number of clusters often critically influences the clustering effect. In the proposed algorithm, authors determine the number of cluster centers based on the generated ρ−δ decision graph or *γ* decision graph. For example, for a dataset with two clusters, its ρ−δ distribution is shown in Fig 4A(cluster centers are marked with a pentagram), and the ρ and *δ* values of the two cluster center points are significantly greater than the values of other data points; thus, these two points can easily be selected as the cluster center. Its *γ* distribution is shown in Fig 4B (cluster centers are marked with a star), and two points with the greatest *γ* value can be selected as cluster centers.

In addition, if the number of clusters is known, this information can be used directly as the input parameter of the algorithm. Note that the algorithm does not need to manually select the cluster center based on the decision graph.

### 4.4 Allocation strategy

The distribution of data points determines the accuracy of the clustering results. In this section, this study introduces a two-step allocation strategy, discuss the similar point concept, present a first-step allocation strategy for similar point, and present a second-step allocation strategy for the remaining unallocated points.

The first step is to assign similar points. After determining the cluster centers according to the decision value and number of clusters, cluster centers are added to a queue. For each element in the queue, the algorithm finds all unallocated similar points, and then classify similar points into the cluster to which the corresponding element belongs. Then, the similar points are added to the end of the queue. Then, the algorithm continues to find similar points of the elements in the queue until there are no unallocated similar points.

The second step is to traverse all k-nearest neighbors of the remaining unallocated points. According to the KNN majority voting strategy, counting the clusters of k-nearest neighbors, and attributing the points to the clusters of most k-nearest neighbors until there are no unallocated points.

In the second step, if there is an unassigned point and its K neighbors are not assigned, or there are as many data points in its K neighbors as belonging to different clusters, the algorithm cannot assign this point through KNN majority voting strategy. Therefore, it is necessary to increase the value of K by 1 until the algorithm can find a certain cluster to which most points in the K nearest neighbor belong and classify the point as this cluster.

### 4.5 Processes

The proposed PFD-DPC algorithm primarily involves two aspects, i.e., (1) calculating the local density, the distance to the nearest higher density points, and the decision value together with (2) determining the cluster centers and using the two-step allocation strategy to allocate data points. The flow of the proposed PFD-DPC algorithm is described as follows.

Algorithm 1

Input: dataset *data*、number of neighbors *K*、diffusion layer *k*

Output: Clustering result *answer*

1. Preprocess the data. Normalize the data and complete missing features.
2. Calculate Euclidean distance matrix *dist*.
3. Calculate the potential field *θ* according to Eq (15).
4. Calculate PFD similarity *Θ* according to Eq (16).
5. Calculate local density *ρ* according to Eq (17).
6. Calculate the distance to the nearest higher density points *δ* according to Eqs (18) and (19).
7. Use *ρ* and δ obtained in steps 5 and 6 to draw a *ρ*−δ decision graph or calculate the decision value γ according to Eq (20) and draw a γ decision graph.
8. In the *ρ*−δ decision graph, select points with greater *ρ and* δ or in the γ decision graph. Then, select points with the greatest γ value as the cluster centers and insert them into queue *Q*.
9. Find unallocated similar points for the element in queue *Q*, classify similar points as the cluster to which the element belongs, and insert the similar points at the end of the queue.
10. Continue to step 9 until there are no unallocated similar points.
11. Traversing k-nearest neighbors for unallocated points. If there are assigned *K* nearest neighbors at this point, then classify this point as the cluster to which most of the allocated k-nearest neighbors belong; otherwise, let *K* → *K*+1.
12. Execute step 11 until there are no unassigned points, at which point the algorithm ends.

### 4.6 Complexity analysis

In this part, this paper analyzes the time complexity and space complexity of the PFD-DPC algorithm.

#### 4.6.1 Time complexity

In this part, this paper will refer to the above algorithm steps, analyze the time complexity of each step, and calculate the time complexity of the whole algorithm. Where n is the number of data points, K is the number of nearest neighbors, k is the diffusion layer, and m is the number of clusters.

In step 1, the attribute values of the data points are completed and normalized, O(*n*).
In step 2, calculate the distance matrix, O(*n*^2^).
In step 3, calculate the Potential field *θ*, O(*Kn*^2^).
In step 4, calculate the PFD similarity *Θ*, O(*K*^*k*^*n*^2^).
In step 5, calculate local density *ρ*, O(*n*^2^).
In step 6, calculate the distance to the nearest higher density points *δ*, O(*n*^2^).
In step 7, the decision graph is drawn using the values of *ρ* and *δ* calculated in steps 5 and 6, which are not included in the main part of the algorithm.
In step 8, manually select the clustering centers according to the decision graph, which is not included in the main part of the algorithm.
In steps 9 and 10, determine the cluster of unallocated similar points, O(*mn*^2^).
In steps 11 and 12, determine the cluster of dissimilar points, O((*K*+*m*)*n*^2^).

Since in the experiment, the value of k ranges from 1 to 3, the value of K ranges from 4 to 50, and the value of m varies from dataset, so it is impossible to determine which *K*^*k*^ or *K*+*m* is larger. For a better representation, this paper takes *M* = *Max*(*K*^*k*^,*K*+*m*), so the time complexity of the PFD-DPC algorithm is O(*Mn*^2^).

#### 4.6.2 Space complexity

The PFD-DPC algorithm needs to use the space size O(*n*^2^) when calculating the distance matrix and the similarity matrix. And storing *ρ* and *δ* only needs the space size of O(*n*), so the space complexity of the PFD-DPC algorithm is O(*n*^2^).

## 5. Experiment

In order to prove the performance of the PFD-DPC algorithm, this paper compared the proposed algorithm to the DPC [19], SNN-DPC [25], FKNN-DPC [33], DBSCAN [9], OPTICS [10], k-means [5], and AP [45] algorithms. The AP, DBSCAN, and k-means algorithms were implemented using the Python sklearn library, and OPTICS used the pyclustering library. Besides, the SNN-DPC algorithm was implemented by the corresponding author’s source code. For the DPC and FKNN-DPC algorithms, the code is reproduced by the algorithm flow described by the author.

### 5.1 Experimental dataset

The performance of the proposed PFD-DPC algorithm was verified using synthetic and real-world datasets. The synthetic and real-world datasets used in the experiments are listed in Tables 1 and 2, respectively (The datasets used in this paper can be downloaded from https://github.com/sdnu-ZhuangHui/Datasets-of-PFD-DPC).

**Table 1:** Synthetic datasets.

Dataset	Source	No records	No attributes	No clusters
Aggregation	[46]	788	2	7
Jain	[47]	373	2	2
Pathbased	[48]	300	2	3
R15	[49]	600	2	15
Spiral	[48]	312	2	3
DIM512	[50]	1024	512	16

**Table 2:** Real-world datasets.

Dataset	Source	No records	No attributes	No clusters
Wine	[51]	178	13	3
WDBC	[51]	569	30	2
Seeds	[51]	210	7	3
Optical Recognition	[51]	5620	64	10
Waveform	[51]	5000	21	3
Ecoli	[51]	336	8	8
Parkinsons	[52]	197	23	2
Dermatology	[51]	366	33	6

### 5.2 Evaluation indicators

In this experiment, three indexes, i.e., adjusted mutual information (AMI) [53], the adjusted Rand index (ARI) [53], and the Fowles Mallows index (FMI) [54] were used to evaluate the performance of the compared clustering algorithms. Note that the upper limit of these indexes is 1, and the closer the value is to 1, the better the clustering effect.

AMI is a measure of the degree of agreement between two datasets. This measure allows us to observe the degree of consistency between the clustering results obtained by a clustering algorithm and the actual categories of the samples. Assuming that the number of samples is *N*, the actual category of the data is *R*, and the clustering result of the data is *C*. AMI is defined as follows.

$$AMI=\frac{MI(R,C)}{\sqrt{H(R)\times H(C)}}$$ (23)

The elements are defined as follows.

$$H(R)=\sum_{i=1}^{\left|U\right|}P(i)log(P(i))$$ (24)

$$H(C)=\sum_{i=1}^{\left|V\right|}P\prime (i)log(P\prime (i))$$ (25)

$$MI(R,C)=\sum_{i=1}^{\left|R\right|}\sum_{j=1}^{\left|C\right|}P(i,j)log(\frac{P(i,j)}{P(i)P'(j)})$$ (26)

$$P(i)=\frac{\left|R_{i}\right|}{N}$$ (27)

$$P(j)=\frac{\left|C_{j}\right|}{N}$$ (28)

The ARI measures the consistency of the distribution of the two datasets. ARI is expressed as follows.

$$ARI=\frac{RI-E(RI)}{max(RI)-E(RI)}$$ (29)

The elements are defined as follows.

$$RI=\frac{a+b}{a+b+c+d}$$ (30)

Here, *a* is the number of data points that belong to the same class in *R* (and belong to the same class in *C*), *b* is the number of data points that do not belong to the same class in *R* (and do not belong to the same class in *C*), *c* is the number of data points belonging to the same class in *R* but not to the same class in *C*, and *d* is the number of data points not belonging to the same class in *R* but belonging to the same class in *C*.

FMI is defined as the geometric mean of the paired accuracy and recall rates.

$$FMI=\frac{a}{\sqrt{\left(a+c)\times (a+d\right)}}$$ (31)

Here, *a*, *c*, and *d* are defined the same as above.

**Fig 5:**
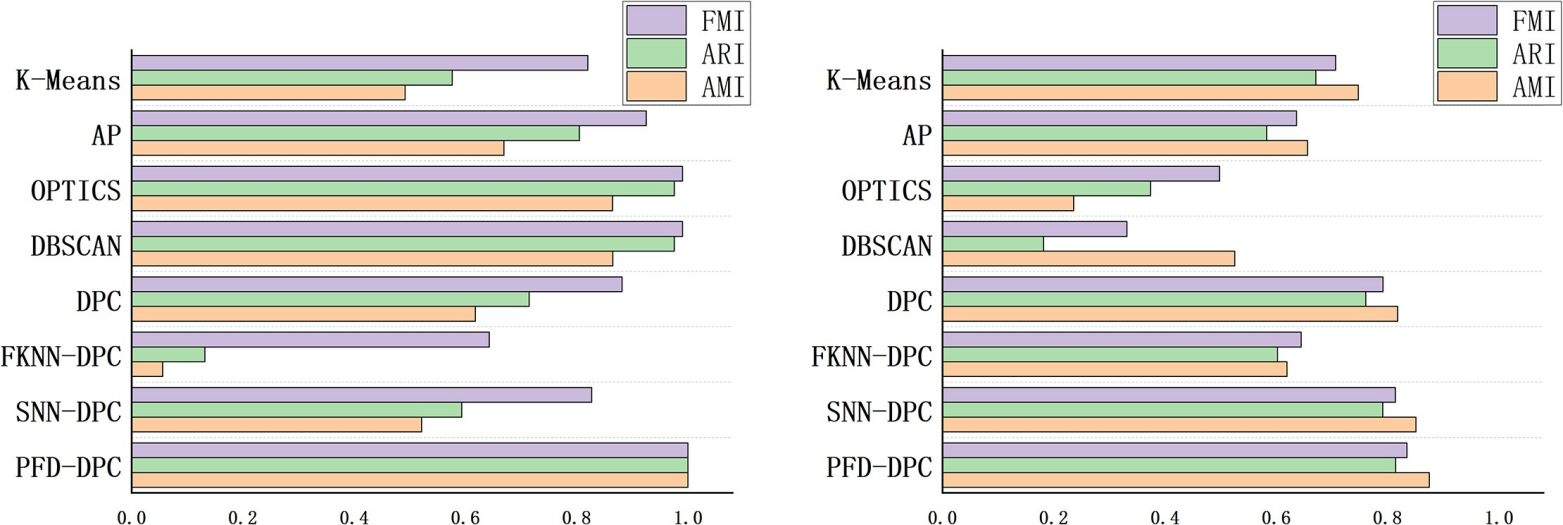
Performance score of each algorithm on datasets. (A) Jain dataset and (B) Optical recognition dataset.

As shown in Fig 5, using the AMI, ARI, and FMI evaluation indicators can intuitively reflect the performance of each algorithm.

### 5.3 Data preprocessing

Prior to performing a clustering experiment, the data must be preprocessed. Data preprocessing primarily includes the completion of missing data and data normalization. In this experiment, the missing eigenvalues were assigned as the mean of the features, and the data were normalized via min–max normalization, which is expressed as follows.

$$x_{ij}=\frac{x_{ij}-\min\left(x_{j}\right)}{\max\left(x_{j}\right)-\min\left(x_{j}\right)}$$ (32)

Here, *i* is the serial number of the sample point, and *j* is the serial number of the feature.

### 5.4 Parameter selection

To evaluate the performance of the algorithms more objectively, authors optimized each algorithm’s parameters.

For the PFD-DPC, SNN-DPC, and FKNN-DPC algorithms, parameter K is required, and this parameter represents the number of nearest neighbors selected and adopts an integer value. Here, authors adopted the value of K as an integer between 4 and 50. Notably, if the value of K is less than the lower limit of 4, the algorithm will fall into a dead cycle, resulting in errors. For the upper limit 50, with an increasing K value, the number of considered neighbor points also increases, the influence of each neighbor gradually decreases, and consequently, the influence of the change on the result gradually decrease. In addition, the PFD-DPC algorithm must specify the diffusion layers *k* of the potential field. Here, *k* is an integer because each layer of diffusion must pay time and space costs; thus, authors adopted *k* values 1 to 3 after weighing the clustering effect and space–time cost.

The cutoff distance *dc* must be set for the DPC algorithm. According to the original author of the DPC algorithm, a *dc* value that makes the number of neighbors account for 1% to 2% of the total number of samples is effective. Therefore, authors adopted a value between 0.1 and 5 with a step size of 0.1 in the experiments.

For the DBSCAN and OPTICS algorithms, two parameters must be set, i.e., the neighborhood radius *ε* and minimum number of samples contained in the neighborhood *minpts*. The neighborhood radius *ε* was set between 0.01 and 1 with a step size of 0.01, and the minimum sample number *minpts* was selected between 1 and 50.

The AP algorithm only has one parameter *preference* to set. For this parameter, large values result in more clustering centers being selected by the algorithm. Here, authors first set a large parameter value, and then gradually narrow the search scope until the best clustering effect is found.

Note that only the correct number of clusters must be specified for the k-means algorithm.

For the PFD-DPC, SNN-DPC, FKNN-DPC, and DPC algorithms, although the cluster center can be selected by a decision diagram, the number of clusters is not always correct; thus, authors specified the correct number of clusters for each algorithm.

### 5.5 Necessity of potential field diffusion

To further verify that the propagation of potential field significantly impacts data point clustering, this paper performed a comparative experiment in which authors only considered potential field without considering diffusion and the diffusion of the potential field.

**Fig 6:**
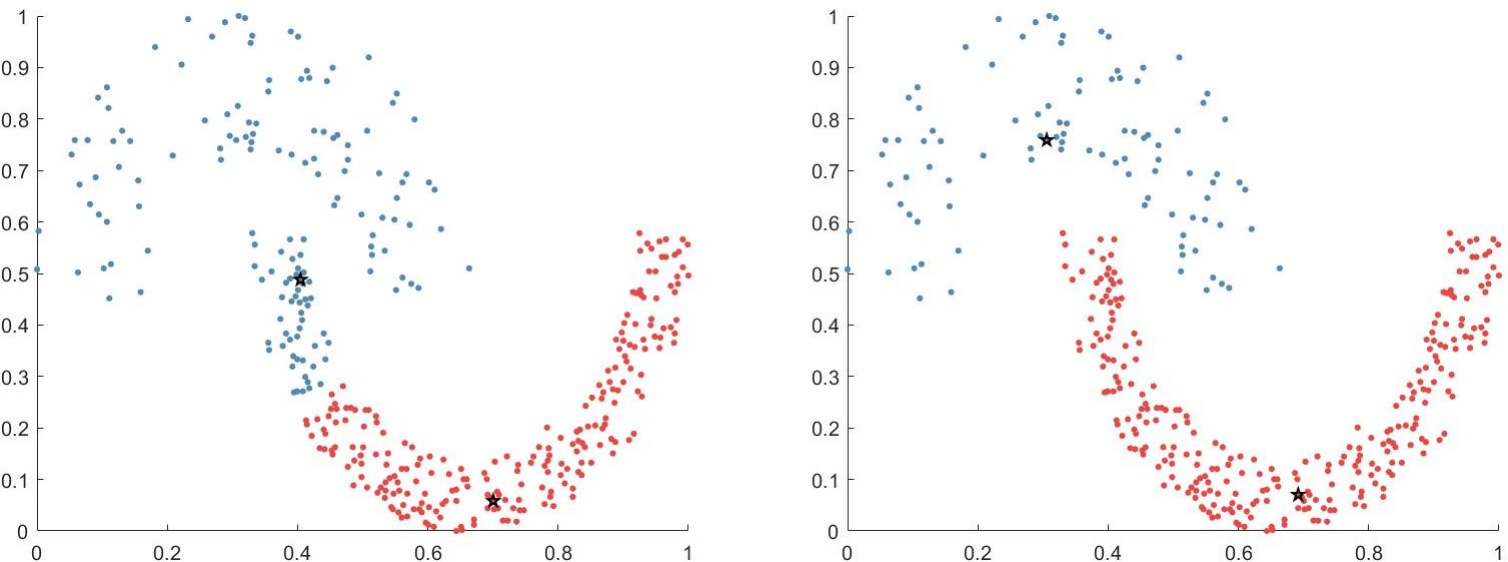
Control experiments on Jain dataset. (A) Regardless of potential field diffusion and (B) Considering potential field diffusion.

Fig 6A shows the clustering results obtained when only the potential field was considered (i.e., diffusion was not considered), and Fig 6B shows the clustering results when the potential field diffusion was considered. Fig 6A shows that, without considering the potential field, the local density of the lower cluster was generally higher than that of the upper cluster owing to the high density of the lower cluster; thus, the two clustering centers were allocated to the lower cluster. In Fig 6B, the diffusion of the potential field was considered in the calculation of PFD similarity. Here, lower cluster density resulted in greater distance from the point to the neighbors during diffusion such that that a cluster with low density will not be ignored.

**Fig 7:**
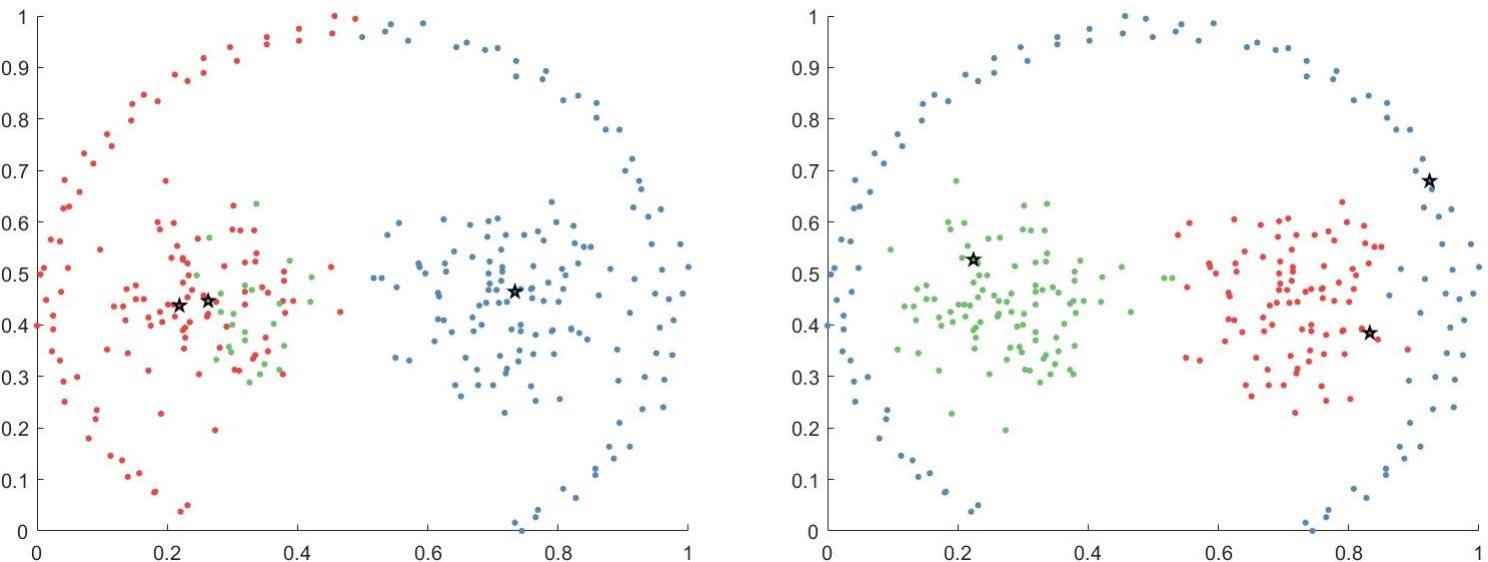
Control experiment on Pathbased dataset. (A) Regardless of potential field diffusion and (B) Considering potential field diffusion.

Fig 7A shows the clustering results obtained when only the potential field was considered (diffusion was not considered), and Fig 7B shows the clustering results when the potential field diffusion was considered. Notably, without considering diffusion, the spherical cluster on the left is divided into two clusters, and the spherical cluster on the right and the right half of the circular cluster are directly classified into the same cluster. However, in Fig 7B, only the individual points on the edge of the cluster generated distribution errors, and the overall clustering effect was far better than that obtained without considering propagation.

These results indicate that the algorithm considering the diffusion of potential field is obviously better than that without considering diffusion because the algorithm that considers the diffusion of potential field pays more attention to the overall distribution of the data points. If only the potential field is considered, the cluster center will be concentrated in the high-density cluster in the variable density cluster, thereby ignoring the low-density cluster, which is undesirable. If the diffusion of the potential field is considered, the distance from the data point to the nearest neighbor point is calculated in each layer of diffusion. The disadvantage of the low-density cluster in the cluster will be changed, thereby making the cluster more reasonable.

### 5.6 Synthetic datasets

This study also experimented on a series of synthetic datasets that are widely used to test various clustering algorithms. These datasets differ in overall distribution, sample size, and number of clusters, which can reflect the performance of an algorithm in different scenarios.

Table 3 shows the clustering results of each algorithm obtained on the synthetic datasets. Here, bold values represent the optimal result of clustering on the dataset. In the following sections, this paper shows the clustering effect of the clustering algorithm on the dataset in the form of pictures, where the star represents the clustering center, the cross represents noise points, and data points in different colors represent different clusters.

**Table 3:** Clustering results on synthetic datasets.

Algorithm	AMI	ARI	FMI	Parameter	AMI	ARI	FMI	Parameter
	Aggregation				Spiral			
PFD-DPC	0.9767	0.9847	0.9880	41/3	**1.0000**	**1.0000**	**1.0000**	5/2
SNN-DPC	0.9803	0.9876	0.9903	6	**1.0000**	**1.0000**	**1.0000**	4
FKNN-DPC	0.9775	0.9855	0.9886	20	**1.0000**	**1.0000**	**1.0000**	5
DPC	**0.9922**	**0.9956**	**0.9966**	3.1	**1.0000**	**1.0000**	**1.0000**	1.8
DBSCAN	0.9529	0.9779	0.9827	0.04/6	**1.0000**	**1.0000**	**1.0000**	0.04/2
OPTICS	0.9221	0.9753	0.9807	0.06/10	**1.0000**	**1.0000**	**1.0000**	0.04/1
AP	0.7667	0.4409	0.5771	-0.50	0.4833	0.1555	0.3469	-0.13
K-Means	0.7935	0.7300	0.7884	7	-0.0055	-0.0060	0.3274	3
	Jain				DIM512			
PFD-DPC	**1.0000**	**1.0000**	**1.0000**	13/3	**1.0000**	**1.0000**	**1.0000**	4/1
SNN-DPC	0.5212	0.5935	0.8272	39	**1.0000**	**1.0000**	**1.0000**	4
FKNN-DPC	0.0562	0.1318	0.6430	10	**1.0000**	**1.0000**	**1.0000**	20
DPC	0.6183	0.7146	0.8819	0.9	**1.0000**	**1.0000**	**1.0000**	0.6
DBSCAN	0.8650	0.9758	0.9906	0.08/2	**1.0000**	**1.0000**	**1.0000**	0.36/2
OPTICS	0.8645	0.9758	0.9906	0.08/1	0.9029	0.9432	0.9478	0.19/1
AP	0.6693	0.8052	0.9253	-1.77	**1.0000**	**1.0000**	**1.0000**	-1.00
K-Means	0.4916	0.5767	0.8200	2	**1.0000**	**1.0000**	**1.0000**	16
	Pathbased				R15			
PFD-DPC	**0.9529**	**0.9707**	**0.9804**	8/1	**0.9938**	**0.9928**	**0.9933**	14/1
SNN-DPC	0.9355	0.9596	0.9730	10	**0.9938**	**0.9928**	**0.9933**	40
FKNN-DPC	0.8344	0.8744	0.9165	9	0.9907	0.9892	0.9899	27
DPC	0.5212	0.4717	0.6664	3.8	**0.9938**	**0.9928**	**0.9933**	0.6
DBSCAN	0.8710	0.9011	0.9340	0.08/10	0.9825	0.9819	0.9831	0.04/12
OPTICS	0.4440	0.6330	0.7486	0.06/4	0.9798	0.9779	0.9794	0.04/11
AP	0.5122	0.4640	0.6635	-4.10	0.9938	0.9928	0.9932	-0.06
K-Means	0.5098	0.4613	0.6617	3	**0.9938**	**0.9928**	**0.9932**	15

**Fig 8:**
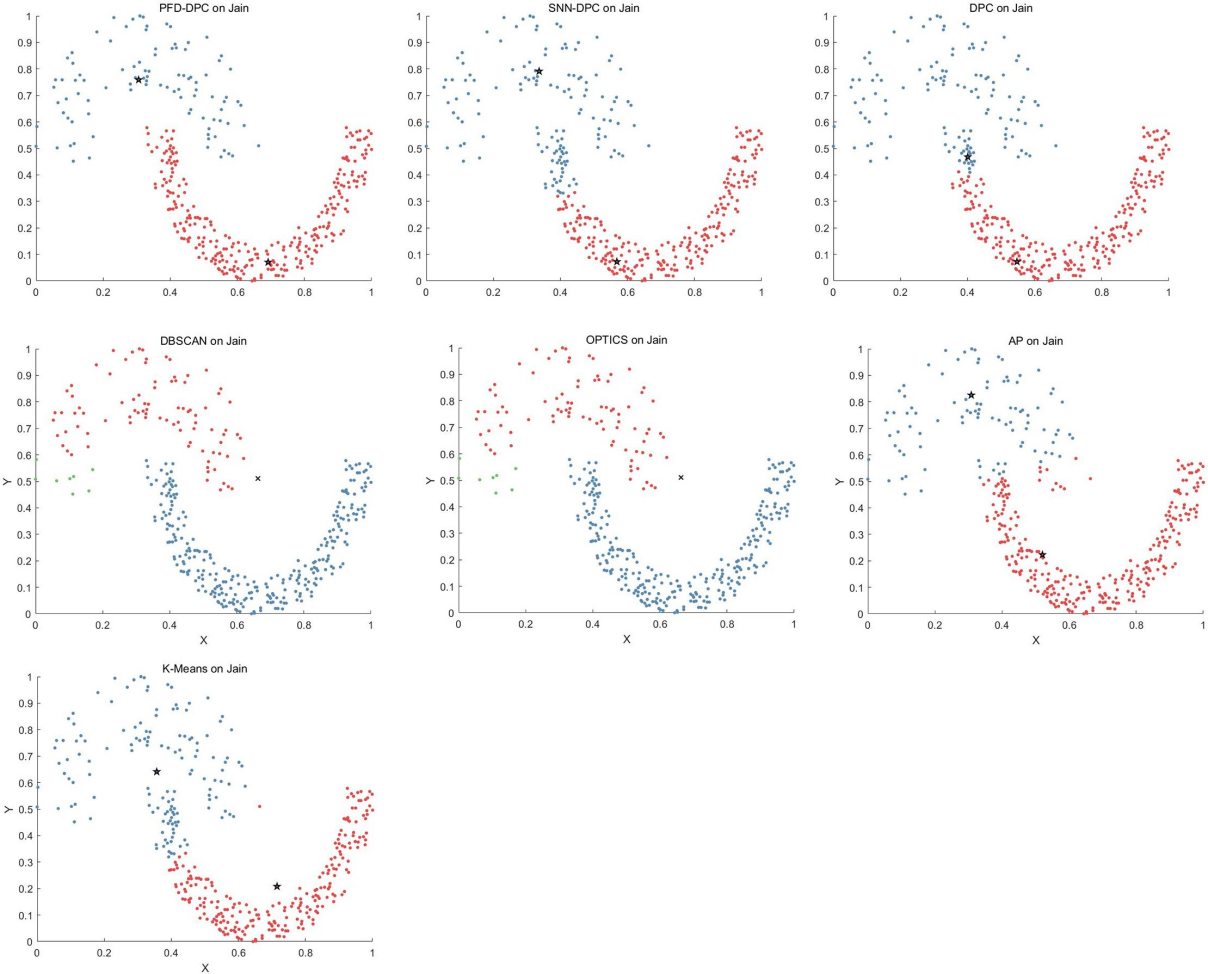
Results of algorithms on Jain dataset.

The clustering effect of each clustering algorithm on the Jain dataset is shown in Fig 8. The Jain dataset comprises two crescent-shaped clusters, in which the density of clusters in the upper left is less than that in the lower right. For the PFD-DPC algorithm, the diffusion of the potential field is considered in the local density calculation; thus, it can better represent the global distribution of the data points rather than relying on traditional local density and distance to the nearest higher density points to determine cluster centers. Therefore, even if the density of clusters in the upper left is small, the algorithm can accurately identify the cluster centers. The SNN-DPC algorithm ignores clusters with lower density at the upper left, and the clusters at the lower right are divided into two different clusters, which is undesirable. With the DPC algorithm, only the number of points within the cutoff distance of the data points is considered when calculating the local density; thus, the local density of clusters with high density is much greater than that of clusters with low density; thus, low-density clusters are ignored. For the DBSCAN and OPTICS algorithms, although the lower right clusters are accurately identified, the upper left clusters are incorrectly divided into two clusters. For the AP and k-means algorithms, cluster opening is still misallocated.

**Fig 9:**
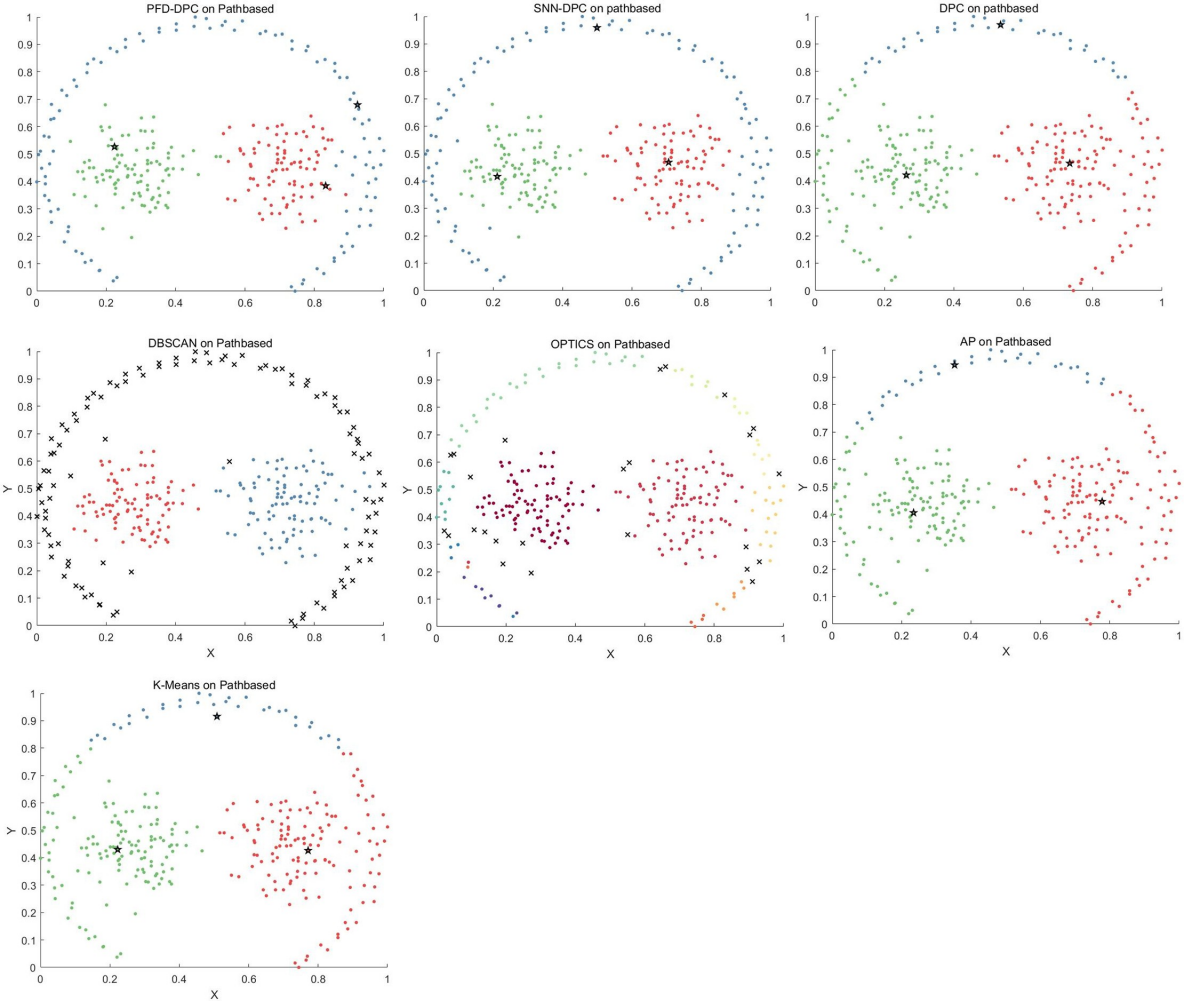
Results obtained on Pathbased dataset.

The clustering effect of each algorithm on the Pathbased dataset is shown in Fig 9. For the PFD-DPC and SNN-DPC algorithms, three clusters could be accurately identified. Although there are a few points on the cluster boundary that produce a few allocation errors, the final clustering effect is still relatively ideal. This is because the PFD-DPC algorithm improves the data allocation strategy and considers its nearest neighbors more reasonably when allocating edge data points, thus reducing the allocation errors. For the DPC, k-means algorithm, and AP algorithms, the data points on both sides were incorrectly allocated to the two clusters in the center, resulting in a series of attached allocation errors. For the DBSCAN algorithm, although two clusters in the center were accurately identified, most points of the peripheral cluster were identified as noise points. For the OPTICS algorithm, although the two clusters in the centers were accurately identified, the points of the peripheral cluster were divided into several different clusters.

**Fig 10:**
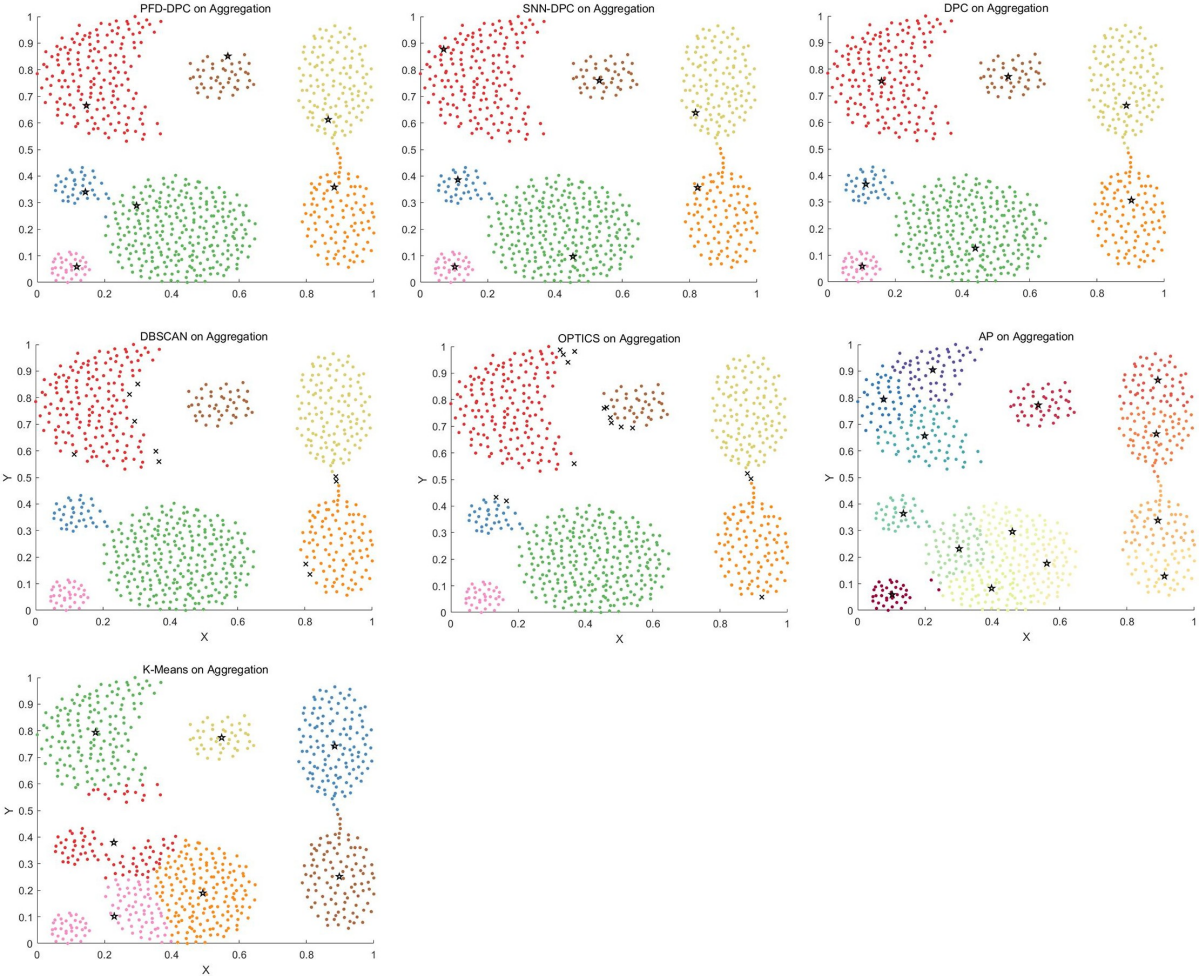
Results obtained on the Aggregation dataset.

The effect of each algorithm on the Aggregation dataset is shown in Fig 10. The density distribution of this dataset is relatively uniform, and the distance of the center of each cluster is relatively far. The traditional DPC algorithm and the improved DPC algorithm assume that the clustering center point has a larger local density and is farther away from the higher density point. Therefore, based on this assumption, the PFD-DPC, SNN-DPC, and DPC algorithms correctly identified the clusters and achieved good results. The DBSCAN and OPTICS algorithms could also correctly identify clusters; however, these algorithms incorrectly evaluated some points on the cluster boundary as noise points. The AP algorithm incorrectly judged the number of clusters, which caused inaccurate clustering. In addition, the k-means algorithm ignored the two smaller clusters on the bottom left and mistakenly divided the two larger clusters into multiple clusters.

**Fig 11:**
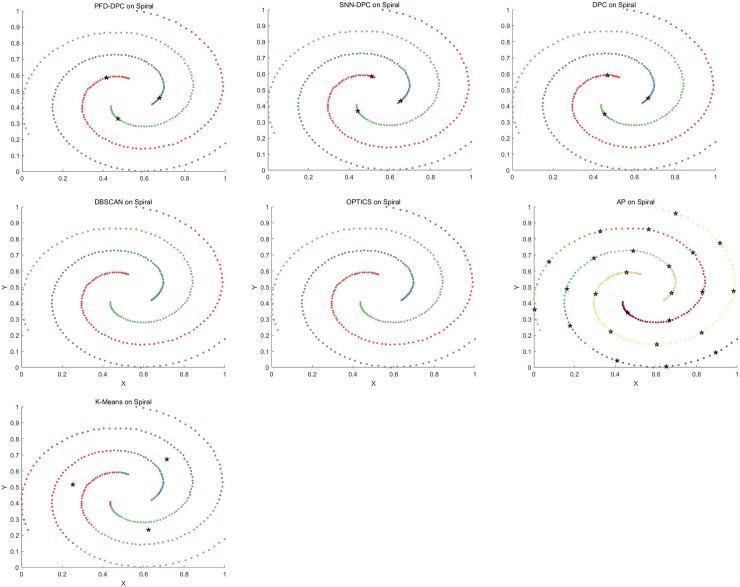
Results obtained on Spiral dataset.

The clustering effect of each algorithm on the Spiral dataset is shown in Fig 11. The PFD-DPC algorithm accurately identified the clusters. This is because after selecting reasonable clustering centers, PFD-DPC algorithm adopts different allocation strategies for similar points and dissimilar points, so that its nearest neighbors are fully considered in the process of data point allocation, thus ensuring the accuracy of clustering. The SNN-DPC, DPC, DBSCAN, and OPTICS algorithms also accurately identified each cluster. The AP algorithm failed to accurately identify the number of clusters, which resulted in serious errors. In addition, the k-means algorithm incorrectly divided samples into three clusters according to the spatial distribution of the data.

In summary, the proposed PFD-DPC algorithm outperformed most of the compared algorithms. Although there were a few allocation errors on the cluster edge of the Aggregation dataset, rendering it slightly inferior to the DPC algorithm, the clustering effect of the proposed PFD-DPC algorithm was obviously superior to the DPC algorithm on the Jain and Pathbased datasets. In addition, the proposed PFD-DPC algorithm was also optimal on the Spiral, R15, and DIM512 datasets.

### 5.7 Real-world datasets

This study compared eight real-world datasets to verify the clustering effect of each algorithm. Note that these datasets differ in data size and structure; thus, it could better verify the performance of each algorithm in different scenarios. Because the dimension of real-world datasets is very high, the picture can not well show the characteristics of the datasets so that this paper will introduce each dataset in detail.

WDBC dataset shows the nuclear digital information of 569 breast masses. Each mass has 30 attributes, and clustering can be used to detect whether the breast mass is benign or malignant [55].

Ecoli dataset consists of 336 protein data of Escherichia coli. It has a total of eight attributes, of which the first attribute is the sequence number, and the remaining seven attributes are calculated from the protein amino acid sequence. The data set can be divided into cp (cytoplasm)、im (inner membrane without signal sequence)、pp (perisplasm)、imU (inner membrane, uncleavable signal sequence)、om (outer membrane)、omL (outer membrane lipoprotein)、imL (inner membrane lipoprotein)、imS (inner membrane, cleavable signal sequence) by clustering.

Seeds dataset consists of 210 wheat kernels. Its seven attributes are area A, perimeter P, compactness C, length of kernel, width of kernel, asymmetry coefficient and length of kernel groove. The dataset can be divided into three categories by clustering.

Dermatology dataset consists of 366 records of patients with skin diseases. Its 33 attributes are the characteristics of clinical evaluation and skin sample evaluation. Through clustering, the dataset can be divided into six categories, which represent different skin diseases.

Parkinsons dataset is composed of a range of biomedical voice measurements from 31 people, 23 with Parkinson's disease, and each attribute is a particular voice measure. Healthy people and patients with parkinson's disease can be identified by clustering.

Optical Recognition dataset consists of 5620 handwritten digital images, its 64 attributes are all integers between 0–16, and the data can be classified into ten types of numbers from 0 to 9 through clustering.

Waveform dataset consists of 5000 sound waveform data, it has 21 attributes with continuous values between 0 and 6, and the dataset can be divided into three categories by clustering.

Wine dataset contains 178 wine records of three different origins, its 13 attributes are the 13 chemical components of wine. Wine of different origins can be classified by clustering.

Table 4 shows the clustering effect of each algorithm on real-world datasets. It can be seen that the PFD-DPC algorithm still performs better than other clustering algorithms on real-world datasets. This is because, on the one hand, the PFD-DPC algorithm introduces the concept of potential field diffusion, comprehensively considering the distance between the data point and its high order neighbors and the nearest higher density point, to determine the clustering center more reasonably; On the other hand, The PFD-DPC algorithm introduces the concept of similar points and adopts a two-step allocation strategy. In the first step, data points are allocated according to similar points. In the second step, the KNN majority voting strategy is used to allocate data points, making the allocation of data points less prone to the attached errors. Therefore, the clustering effect of the PFD-DPC algorithm on real-world datasets is superior to other clustering algorithms, proving the superiority of the PFD-DPC algorithm.

**Table 4:** Clustering results on real-world datasets.

Algorithm	AMI	ARI	FMI	Parameter	AMI	ARI	FMI	Parameter
	WDBC				Ecoli			
PFD-DPC	**0.7433**	**0.8438**	**0.9274**	30/2	**0.6458**	**0.7481**	**0.8252**	14/1
SNN-DPC	0.7350	0.8373	0.9244	30	0.5463	0.6794	0.7840	25
FKNN-DPC	0.3560	0.4009	0.7658	9	0.4755	0.5535	0.6919	9
DPC	0.0007	-0.0028	0.7257	1.3	0.4978	0.4465	0.5775	0.4
DBSCAN	0.3581	0.4786	0.7570	0.46/38	0.4516	0.5255	0.6623	0.20/22
OPTICS	0.0856	0.4305	0.6767	0.51/65	0.4260	0.6642	0.7515	0.23/29
AP	0.5936	0.1322	0.7879	262	0.5339	0.4907	0.6134	-0.86
K-Means	0.6110	0.7302	0.8770	2	0.5051	0.4190	0.5542	8
	Seeds				Dermatology			
PFD-DPC	**0.7600**	**0.8093**	**0.8723**	38/1	**0.9286**	**0.9278**	**0.9422**	25/1
SNN-DPC	0.7574	0.7987	0.8653	6	0.8749	0.8689	0.9021	19
FKNN-DPC	0.6971	0.7422	0.8276	9	0.8355	0.8127	0.8504	35
DPC	0.7299	0.7670	0.8444	0.7	0.7470	0.6893	0.7512	2.2
DBSCAN	0.5302	0.5291	0.6711	0.24/16	0.5721	0.4165	0.5395	0.99/3
OPTICS	0.3802	0.4190	0.6350	0.81/5	0.2934	0.3430	0.4563	0.99/1
AP	0.4465	0.3936	0.6933	-2.07	0.6898	0.5935	0.6766	-0.84
K-Means	0.6705	0.7049	0.8026	3	0.8748	0.7426	0.7947	6
	Parkinsons				Waveform			
PFD-DPC	0.1772	**0.2686**	**0.8140**	43/3	0.3821	0.3016	0.5356	4/3
SNN-DPC	0.2127	0.1187	0.6150	21	**0.3983**	**0.4176**	**0.6164**	7
FKNN-DPC	0.1336	0.1601	0.6582	7	0.0774	0.0086	0.5050	6
DPC	**0.2478**	0.1256	0.6187	1.2	0.3261	0.2698	0.5292	0.1
DBSCAN	0.0071	0.0252	0.5775	0.50/17	0.0856	0.0097	0.4813	0.38/5
OPTICS	0.0368	0.0986	0.5049	0.45/9	0.0286	0.0918	0.2661	0.47/48
AP	0.1098	0.0343	0.2246	0.23	0.2891	0.3014	0.5178	-2.20
K-Means	0.2129	0.0520	0.5957	2	0.3630	0.2536	0.5037	3
	Optical Recognition				Wine			
PFD-DPC	**0.8751**	**0.8146**	**0.8350**	31/3	**0.9081**	**0.9295**	**0.9532**	36/1
SNN-DPC	0.8514	0.7916	0.8140	28	0.8928	0.9121	0.9417	38
FKNN-DPC	0.6195	0.6026	0.6452	10	0.8038	0.7990	0.8667	9
DPC	0.8182	0.7612	0.7920	0.3	0.7065	0.6724	0.7835	2.0
DBSCAN	0.5254	0.1817	0.3318	0.99/3	0.5484	0.5292	0.7121	0.50/21
OPTICS	0.2358	0.3741	0.4983	0.99/1	0.3698	0.4119	0.6296	0.59/7
AP	0.6566	0.5829	0.6365	-212.92	0.3330	0.3170	0.6126	-2.02
K-Means	0.7475	0.6712	0.7070	10	0.8473	0.8685	0.9126	3

According to the clustering effect of the PFD-DPC algorithm on real-world datasets, it can be concluded that the PFD-DPC algorithm can be applied to recognize the handwritten digits, diseases, wine and so on.

By comparing the synthetic datasets with the real-world datasets, it can be found that all the clustering algorithms in this paper are less effective on the real-world datasets than on the synthetic datasets, because the real-world datasets often have a very high dimension. However, when authors calculate the distance matrix of data points, they often use Euclidean distance. According to [56], high-dimensional Euclidean distance loses almost all its meaning, so the clustering effects of clustering algorithms on real datasets are often worse than those on synthetic datasets.

## 6. Discussion

In this part, this paper analyzes the parameter sensitivity, data sequence sensitivity and running time of the PFD-DPC algorithm.

### 6.1 Parameter sensitivity analysis

In this section, this paper analyzes the parameter sensitivity of the PFD-DPC algorithm.

The PFD-DPC algorithm has two input parameters, K and k, where K represents the nearest neighbor, and k represents the number of diffusion layers. The experimental part above has given the optimal parameters of the PFD-DPC algorithm on each dataset. The authors select some representative datasets and change the value of K to carry on the experiment when the k value is determined to be the optimal value.

**Fig 12:**
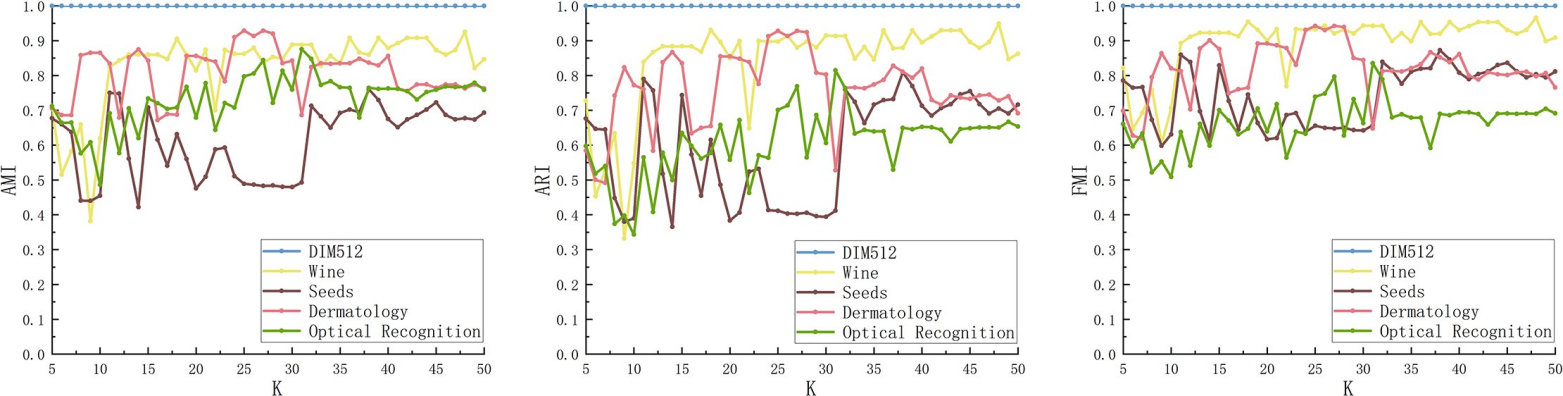
AMI (a), ARI (b), FMI (c) of PFD-DPC algorithm under different K values.

The AMI, ARI and FMI values of the PFD-DPC algorithm under different K values are shown in Fig 12. It can be seen that the three measures fluctuate greatly when the value of K is small, but with the increase of the value of K, the values of the three measures are gradually stable. For most datasets, the values of AMI, ARI, and FMI will be vigorously jittered before K = 33, and then they will stabilize. Therefore, in order to obtain a relatively stable clustering effect, when selecting the K value, a value greater than or equal to 33 can be selected to ensure that the PFD-DPC algorithm is robust.

### 6.2 Data sequence sensitivity analysis

In this section, this paper analyzes the data sequence sensitivity of the PFD-DPC algorithm.

**Fig 13:**
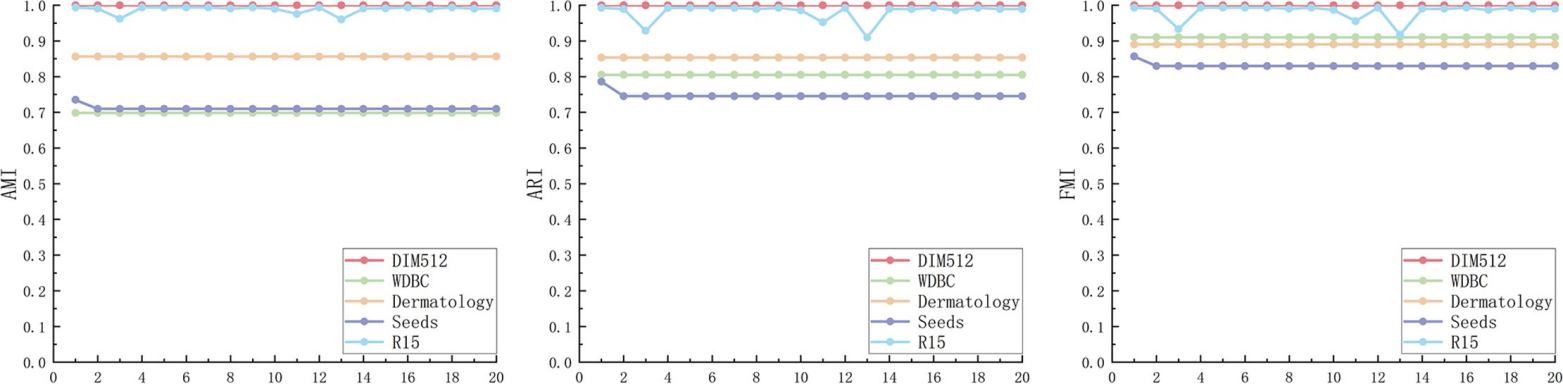
AMI (a), ARI (b) and FMI (c) of PFD-DPC algorithm under random data sequence.

The authors selected several representative datasets and let the PFD-DPC algorithm cluster each dataset under the optimal parameters. Before each clustering, the authors randomly disrupts the data sequence of each dataset and carries out 20 experiments for each dataset. The values of AMI, ARI and FMI of the PFD-DPC algorithm in random data sequence are shown in Fig 13. It can be seen that the three measures of the algorithm tend to be stable on most datasets, and only the measures of individual experiments will fluctuate, but there is no sharp fluctuation. Therefore, it can be concluded that the PFD-DPC algorithm is not sensitive to the sequence of data.

### 6.3 Running time

The running time of the algorithm is an important criterion to evaluate the quality of the algorithm. In this section, this paper will compare the running time of the PFD-DPC algorithm with the traditional DPC algorithm. According to [18], the time complexity of the DPC algorithm is *O*(*n*^2^). And this paper has calculated that the time complexity of the PFD-DPC algorithm is O(*Mn*^2^). Although the time complexity of PFD-DPC is M times that of the DPC algorithm, the actual running time of the algorithm does not have such a big gap.

This paper compares the running time of the PFD-DPC algorithm and the DPC algorithm on each dataset. In order to reduce the errors of the experiment, this paper runs the two algorithms 50 times under the optimal parameters, and compares the average time required for the algorithm to run once. The results are shown in Table 5. It can be seen that on most datasets, the running time of the PFD-DPC algorithm and DPC algorithm is not much different, but the clustering effect of the PFD-DPC algorithm on most datasets is better than DPC algorithm.

**Table 5:** Running time of the algorithms on different datasets (in seconds).

Dataset	PFD-DPC	DPC
Aggregation	0.5491	0.0642
Spiral	0.0754	0.0500
Jain	0.1303	0.0221
DIM512	4.2478	3.4751
Pathbased	0.0667	0.0101
R15	0.2958	0.0251
WDBC	2.7401	2.6005
Ecoli	1.3955	1.4497
Seeds	0.8393	0.8773
Dermatology	1.6414	1.4608
Parkinsons	0.8127	0.7495
Waveform	40.1716	29.2842
Optical Recognition	61.0278	49.8040
Wine	0.7281	0.6761

## 7. Conclusion

Considering the shortcomings of the DPC algorithm, this study proposed the PFD-DPC algorithm that defines a new density measure based on the diffusion of the potential field, rendering the selection of clustering centers more reasonable and enabling a more accurate determination of the cluster centers. In addition, the algorithm redefines the judgment conditions of similar point, improves the reliability of data allocation, and prevents the occurrence of attached errors during data point allocation; thus, serious clustering errors can be avoided. The experimental results obtained on both synthetic and real-world datasets demonstrate that the proposed PFD-DPC algorithm can be applied to a dataset with significant density difference, cross winding, and high feature latitude between clusters.

In the future, the authors plan to focus on three aspects. First, they will examine how the proposed PFD-DPC algorithm can automatically, quickly, and accurately determine cluster centers rather than artificially specifying the number of clusters. Second, they plan to investigate how to adaptively determine the layer of diffusion of the potential field and automatically determine reasonable layers for clusters with different structures as well as reduce the time and space costs while ensuring sufficient clustering accuracy. Third, they will investigate methods to reduce the computational complexity of the proposed PFD-DPC algorithm while maintaining its superior clustering effect.

## Supporting information

S1 Data (ZIP)
